# Machine Learning Prediction and Reducing Overdoses With Electronic Health Record Nudges (mPROVEN) in the Primary Care Setting: Protocol for a Cluster Randomized Controlled Trial

**DOI:** 10.2196/94007

**Published:** 2026-05-04

**Authors:** Walid F Gellad, Yi-Fan Chen, Tae Woo Park, Qingnan Yang, Jonathan D Arnold, Courtney C Kuza, Stephanie N Fedro-Byrom, Julie Diiulio, Laura G Militello, Michelle Whitlock, Eugene M Sadhu, Shyam Visweswaran, Michael J Fine, Kaleab Z Abebe, Katie J Suda, Wei-Hsuan Lo-Ciganic

**Affiliations:** 1 Division of General Internal Medicine University of Pittsburgh Pittsburgh, PA United States; 2 Center for Pharmaceutical Policy and Prescribing University of Pittsburgh Pittsburgh, PA United States; 3 VA Pittsburgh Healthcare System Pittsburgh, PA United States; 4 Center for Biostatistics and Qualitative Methodology Division of General Internal Medicine University of Pittsburgh Pittsburgh, PA United States; 5 Department of Psychiatry University of Pittsburgh Pittsburgh, PA United States; 6 Applied Decision Science Cincinnati, OH United States; 7 Department of Biomedical Informatics University of Pittsburgh Pittsburgh, PA United States; 8 Geriatric Research Education and Clinical Center North Florida/South Georgia Veterans Health System Gainesville, FL United States

**Keywords:** cluster randomized trial, opioid, machine learning, risk prediction, behavioral nudge, best practice alert, electronic health record platforms, primary care, opioid overdose

## Abstract

**Background:**

Opioid overdose remains a leading cause of preventable death in the United States. Existing approaches to identify individuals at elevated risk rely on imprecise rule-based criteria that misclassify patients’ risk of this serious health outcome. Machine learning (ML) algorithms can help improve prediction performance and can be combined with electronic health record (EHR) interventions to reduce overdose risk.

**Objective:**

The Machine Learning Prediction and Reducing Overdoses With EHR Nudges (mPROVEN) clinical trial integrates a validated ML overdose risk model with behavioral economics–informed EHR nudges to test whether the combination improves evidence-based prescribing behaviors associated with lower overdose risk and, ultimately, reduces overdose among elevated-risk patients.

**Methods:**

mPROVEN is a pragmatic cluster randomized controlled trial conducted in primary care practices within a large multistate integrated health system. Eligible patients are adults (≥18 years) identified by the ML algorithm as having elevated overdose risk and seen at a primary care visit during the study period. Primary care practices serve as the unit of randomization and will be randomized into three arms: (1) usual care; (2) elevated risk flag only, where clinicians see a noninterruptive EHR flag indicating elevated overdose risk; and (3) elevated risk flag + nudges, in which active choice and accountable justification alerts are embedded within the EHR in addition to the elevated risk flag. The trial will enroll a target cohort of 800 patients for the primary analysis. The intervention period is 4 months (or until the study ends, whichever occurs later). The primary outcome is a 3‑point composite measure of safer opioid prescribing at 4 months, awarding 1 point each for active naloxone prescription, average opioid dosage of 50 morphine milligram equivalents per day or less, and absence of opioid-benzodiazepine overlap. Secondary outcomes include the composite outcome at 6 months, individual score components, and all-cause and overdose-specific emergency department or inpatient visits. Outcomes will be compared across study arms using an intention‑to‑treat approach with linear mixed‑effects models accounting for clinic-level clustering.

**Results:**

Funded by the National Institutes of Health, in June 2022, enrollment began on March 10, 2025. Enrollment for the primary analysis cohort (n=798) was completed in May 2025 with additional participants enrolled for secondary analyses through December 2025 (n=1662). Primary cohort analyses began in January 2026, and results are expected by mid-2027.

**Conclusions:**

The mPROVEN study is among the first pragmatic randomized controlled trials to integrate ML‑based opioid overdose risk prediction with behavioral nudges within a large health system EHR. By combining advances in data science and behavioral economics, the study aims to reduce opioid overdose risk in primary care using a scalable and low-touch intervention to address a high-priority public health issue.

**Trial Registration:**

ClinicalTrials.gov NCT06806163; https://clinicaltrials.gov/study/NCT06806163

**International Registered Report Identifier (IRRID):**

DERR1-10.2196/94007

## Introduction

Opioid overdose remains a leading cause of preventable death in the United States, with 54,743 opioid‑related deaths in 2024 [1]. The economic and societal burden of opioid use disorder (OUD) and fatal opioid overdose exceeds US $1 trillion annually, including health care expenditures, lost productivity, premature mortality, reduced quality of life, and criminal justice involvement [2].

Although health systems and other stakeholders have instituted multiple interventions to reduce patient risk of overdose [3,4], including promoting safer opioid prescribing and dispensing naloxone, such efforts face 2 noteworthy challenges. First, there are limited and poorly performing tools to help clinicians identify who is truly at risk of overdose, leading to imprecise and burdensome interventions that target an overly broad population [5,6] or fail to identify the high-risk individuals most likely to experience overdose. Second, even with more accurate identification of elevated-risk patients, highly effective strategies to change clinician therapeutic behaviors remain limited.

To address these challenges, our team previously developed and externally validated machine learning (ML) algorithms for overdose prediction. In an application in Medicare data, these algorithms identified more than 90% of overdoses among the top risk groups, whereas commonly used risk classifications such as the high opioid dose measure used by the Centers for Medicare and Medicaid Services missed 70% to 90% of individuals with an actual overdose [3,7,8]. However, even with more accurate risk prediction in clinical settings, prognostic information may not translate into clinical action because clinicians face workflow challenges, biases and clinical inertia, and other barriers [9,10]. Behavioral nudges, which are subtle changes to how choices are presented or how information is framed to influence behavior without restricting choice, can help facilitate action by clinicians [11-16]. One nudge strategy, the *accountable justification*, prompts clinicians to briefly document a rationale for a clinical decision, and it has increased evidence-based oncology prescribing, reduced unnecessary antibiotic use, and decreased opioid prescribing in prior studies [17-19].

The Machine Learning Prediction and Reducing Overdoses With Electronic Health Record (EHR) Nudges (mPROVEN) study combines more accurate ML risk prediction with behavioral nudges as a targeted intervention to improve prescribing practices associated with overdose risk and, ultimately, reduce overdose risk itself. We integrated a validated ML-driven opioid overdose risk prediction model into a large health system’s EHR to identify patients at elevated risk of opioid overdose. We will conduct a 3-arm pragmatic, cluster randomized controlled trial (RCT) among patients with an elevated predicted risk of overdose, delivering within the EHR risk information, active choice alerts, and accountable justification alerts to primary care providers who see elevated-risk patients.

## Methods

### Overview and Study Setting

We designed mPROVEN as a 3-arm pragmatic cluster RCT conducted in primary care practices within the UPMC health system. UPMC is an integrated health system that includes 181 primary care (internal medicine and family medicine) practices in urban and rural settings in western and central Pennsylvania, New York, and northern Maryland. All outcomes and measures will be obtained from the UPMC health system EHR (Epic). This project is registered at ClinicalTrials.gov (identifier: NCT06806163). This trial adheres to the CONSORT (Consolidated Standards of Reporting Trials) extension for pragmatic trials [20] and aligns with the Pragmatic Explanatory Continuum Indicator Summary 2 framework for pragmatic study design [21].

### ML Opioid Overdose Risk Prediction Model

**Figure 1:**
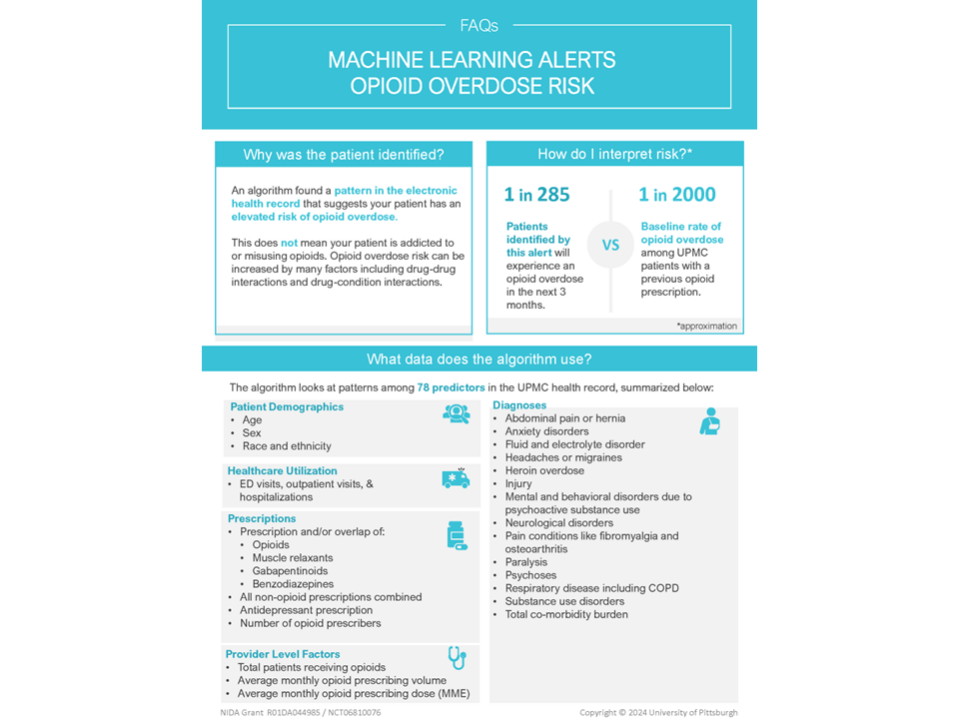
First page of the Machine Learning Prediction and Reducing Overdoses With Electronic Health Record Nudges (mPROVEN) frequently asked questions (FAQ) document.

The ML risk prediction model uses a gradient boosting algorithm to predict 3‑month overdose risk using EHR data (eg, demographic characteristics, diagnosis codes, prescription orders and fills, and clinical encounters) [7,8,11,22]. Eligible primary care patients (defined below) have a risk score calculated weekly, and those whose predicted risk exceeds a predefined threshold (ie, ≥92.5th percentile in the training cohort) are classified as elevated risk. Model fairness and calibration were evaluated during development and further confirmed during a 4-month silent testing period conducted in the background of the UPMC Epic system. To support clinician understanding of the algorithm, we created a “frequently asked questions” (Figure 1) document embedded in the clinical trial alerts that summarizes model details, misclassification rates, and key limitations in an accessible format.

### Description of Interventions: Elevated Risk Alert Flags and Behavioral Nudges

#### Elevated Risk Alert Flag

**Figure 2:**
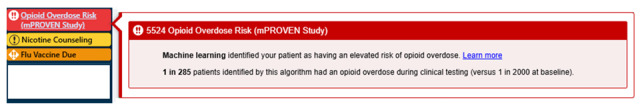
Machine Learning Prediction and Reducing Overdoses With Electronic Health Record Nudges (mPROVEN) storyboard alert (copyright Epic Systems Corporation 2026; reproduced with permission from the copyright holder).

Clinicians in the intervention arms receive a clinical alert indicating that a patient is at elevated risk of opioid overdose. The “elevated risk flag” is a noninterruptive alert displayed in the “storyboard” section of the Epic EHR, where it is visible to clinicians during the encounter. Additional information becomes visible when the clinician hovers over the alert (Figure 2). The storyboard alert will be present at each encounter while the study is active if the patient continues to meet the elevated risk criteria.

#### Behavioral Nudges

In addition to the elevated risk flag, clinicians in the elevated risk flag and behavioral nudges arm can also receive 2 types of EHR-embedded behavioral nudges: active choice and accountable justification.

##### Active Choice Nudge (Naloxone Ordering Prompt)

**Figure 3:**
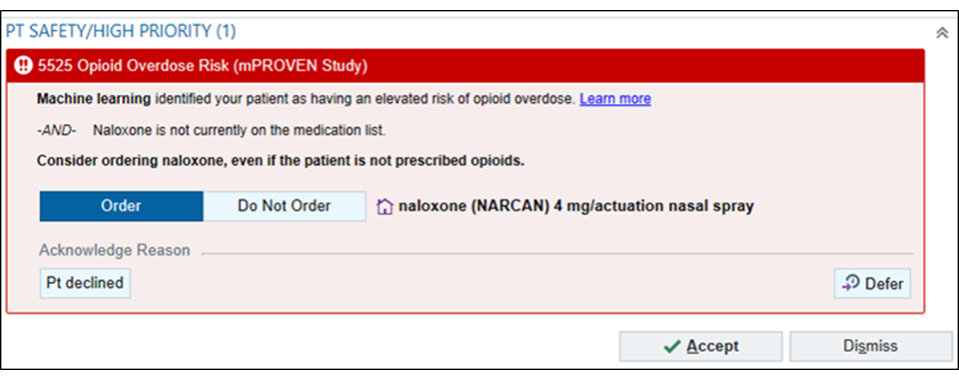
Machine Learning Prediction and Reducing Overdoses With Electronic Health Record Nudges (mPROVEN) naloxone prescription behavioral nudge (copyright Epic Systems Corporation 2026, reproduced with permission from the copyright holder).

The active choice nudge will prompt clinicians to prescribe naloxone when caring for an elevated-risk patient who does not have naloxone on their medication list and who has not had a naloxone order in the previous year. The alert (Figure 3) will appear whenever any prescription order is entered, with the default option to prescribe naloxone.

Clinicians have several options to address this alert: (1) select “Accept” to order naloxone, (2) toggle “Do Not Order” and select “Accept” to not order naloxone; (3) select “Pt declined” to document that the patient declined naloxone, (4) select “Dismiss” to have the order go away (it will reappear if the eligibility criteria are met at the next visit), or (5) select “Defer” to temporarily move the alert to the storyboard for 24 hours. If naloxone is not ordered and the clinician does not use the “patient declined button,” the alert will reappear when an order is placed again in another encounter.

##### Accountable Justification Nudges

**Figure 4:**
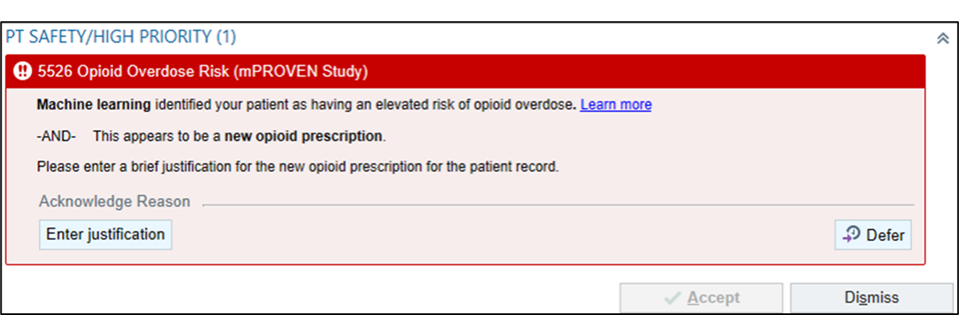
Machine Learning Prediction and Reducing Overdoses With Electronic Health Record Nudges (mPROVEN) new opioid accountable justification nudge alert (copyright Epic Systems Corporation 2026, reproduced with permission from the copyright holder).

**Figure 5:**
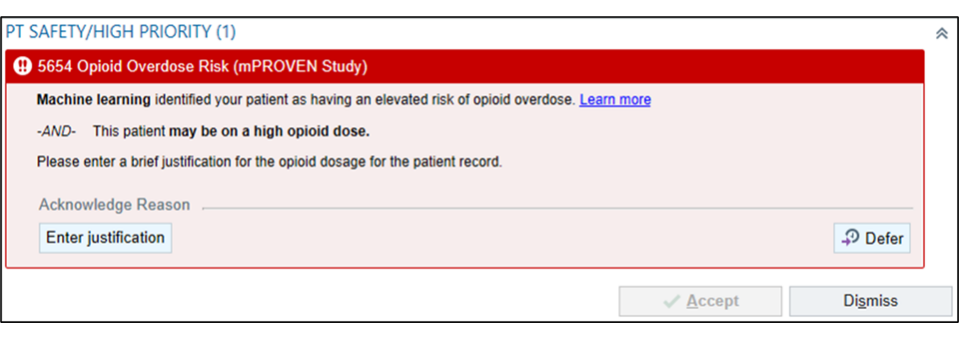
Machine Learning Prediction and Reducing Overdoses With Electronic Health Record Nudges (mPROVEN) high dosage accountable justification nudge alert (copyright Epic Systems Corporation 2026, reproduced with permission from the copyright holder).

**Figure 6:**
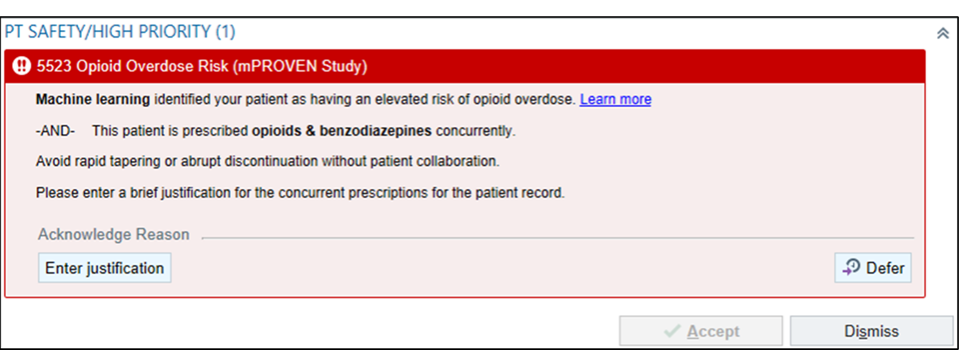
Machine Learning Prediction and Reducing Overdoses With Electronic Health Record Nudges (mPROVEN) opioid and benzodiazepine overlap accountable justification nudge alert (copyright Epic Systems Corporation 2026, reproduced with permission from the copyright holder).

The accountable justification nudges appear for elevated-risk patients in the intervention arm when clinicians perform any of the following prescribing actions in the EHR, which are associated with an elevated risk of overdose [23]: (1) initiate a new opioid prescription for a patient who has had no opioid prescribed in the previous year (Figure 4), (2) order an opioid dose greater than 50 morphine milligram equivalents (MMEs) based on Epic’s automated opioid dosage calculation (Figure 5), and/or (3) prescribe concomitant opioids and benzodiazepines when one medication is prescribed while the other remains active on their medication list (Figure 6).

These 3 justification nudges have parallel functionality. For each, clinicians are prompted to enter a brief rationale into the medical record explaining the prescribing decisions for these elevated-risk patients. Clinicians may also “dismiss” the alert for the encounter or “defer” it until later in the same encounter. If the clinician does not enter a justification or dismisses the alert, it will reappear at the next eligible encounter (eg, a subsequent visit where an opioid and benzodiazepine are coprescribed). If a justification is entered, the alert will remain silent for 3 months and reappear only if the patient meets the triggering criteria.

### Pilot-Testing of the mPROVEN Intervention

**Figure 7:**
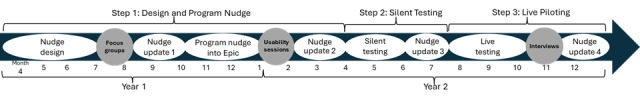
Timeline and steps for the Machine Learning Prediction and Reducing Overdoses With Electronic Health Record Nudges (mPROVEN) silent and live testing.

The pilot phase of mPROVEN study was conducted over a 2-year period and consisted of 3 sequential steps (Figure 7). This approach emphasized close partnership with end users and other stakeholders to guide prioritization and iterative development cycles with frequent updates. We partnered with human factors experts to draft the initial design of the nudges.

To gather end user feedback on the nudge designs, we conducted 4 clinician focus group sessions centered on the nudges’ content, presentation, and workflow integration. Feedback from these sessions informed revisions to the nudge designs. After the revised nudges were programmed into an Epic test environment, we held 6 one-on-one usability sessions with clinicians using an iterative feedback process to refine the nudges’ interface and functionality. Following usability refinements to the nudge intervention based on early focus groups, we initiated a 4-month silent testing phase in which the nudges described above ran *in the background* of the EHR across all UPMC primary care practices for elevated-risk patients without being visible to clinicians. This phase allowed the research team to validate the technical performance of the nudges, monitor alert volumes by practice, and ensure that the data infrastructure for outcome measurement functioned as intended. After confirming operational readiness, we proceeded to a 4-month live testing phase in 2 primary care practices. During this phase, clinicians were exposed to the elevated risk flag and the nudges during patient encounters. We collected quantitative data on clinician engagement with the nudges and qualitative feedback through a round of interviews. Insights from the live testing phase were used to finalize the intervention and prepare for the launch of the clinical trial.

### Trial Design

**Figure 8:**
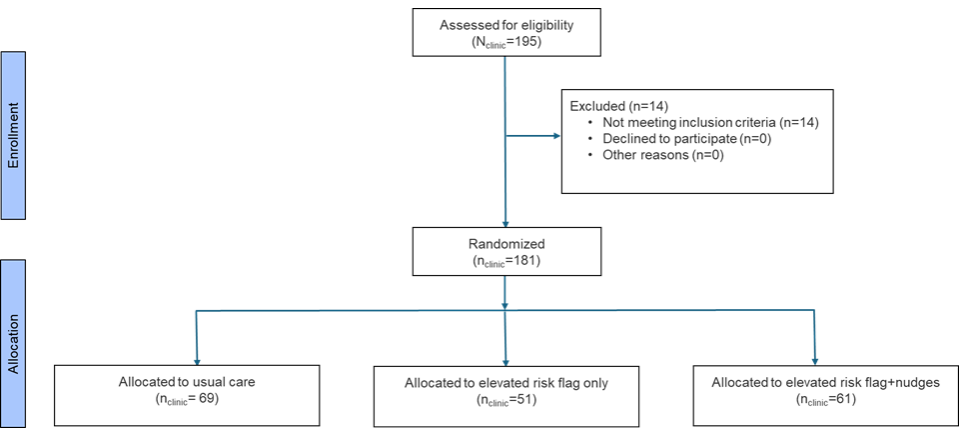
CONSORT (Consolidated Standards of Reporting Trials) [20] flow diagram of the Machine Learning Prediction and Reducing Overdoses With Electronic Health Record Nudges (mPROVEN) cluster randomized controlled trial across 181 primary care practices in the UPMC health system.

UPMC primary care practices serve as the unit of randomization, and all clinicians within a randomized practice receive the intervention assigned to the practice (Figure 8 [20]). Practices will be randomized to three arms: (1) usual care; (2) elevated risk flag only, where clinicians see a noninterruptive EHR flag indicating that a patient meets the elevated risk threshold; and (3) elevated risk flag + nudges, where clinicians see the elevated risk flag plus active choice and accountable justification alerts embedded within the EHR.

This design allows us to assess the incremental benefit of adding behavioral nudges to an elevated risk flag and compare each intervention with usual care. Elevated-risk patients are enrolled into the trial at the time of the first qualifying primary care visit when one of the trial alerts is triggered (or, if in the usual care group, when an alert would have been triggered).

### Study Participants and Enrollment

Patients are eligible for enrollment in the trial if they are aged 18 years or older, have had an opioid prescription in the prior year, have an office or telemedicine primary care visit at a participating practice, and are identified as being at elevated risk by the ML algorithm. Patients are excluded if they had a malignant cancer diagnosis in the prior year or are receiving hospice care; if individuals develop a new cancer diagnosis or receive hospice care after enrollment, they will no longer receive the intervention. Importantly, although model development was restricted to patients receiving an opioid prescription in the prior year, the algorithm generates accurate risk estimates regardless of whether a patient has an active opioid prescription at the time of risk scoring.

### Study Outcomes

All trial outcomes and measures will be derived from the UPMC health system EHR. Follow-up for each patient begins at the date of enrollment (first encounter of an elevated-risk patient with a practice clinician). The primary outcome will be assessed 4 months after each eligible patient’s first encounter during the study period. This 4-month period was revised from the originally planned 6-month outcome (which is now a secondary outcome) due to an unexpected EHR system transition in September 2025.

The primary outcome is a composite measure of 3 prescribing practices scored as a 3-point ordinal score (0-3) in which each component contributes 1 point. A higher score indicates prescribing patterns associated with a lower overdose risk. Patients receive 1 point for each of the following: (1) an active naloxone prescription, defined as a naloxone fill or order within the previous year; (2) an opioid dosage of less than 50 MME per day, calculated as the average daily MME over the preceding 7 days based on prescription fill data or order data; and (3) the absence of overlapping opioid and benzodiazepine prescriptions, defined as no concurrent opioid and benzodiazepine fills or orders within the last 28 days.

For components 2 and 3, individuals not receiving any opioid prescriptions receive 1 point for each of those components. A maximum composite score of 3 is achieved when a patient (1) has an active naloxone prescription, (2) is not receiving high-dose opioids (≥50 MME), and (3) does not have concurrent benzodiazepine and opioid prescriptions within the previous 28 days. We *hypothesize* that our intervention will result in safer prescribing behaviors among elevated-risk patients, reflected by higher composite scores. Because overdose events are relatively rare within the trial time frame, the study is not powered to detect differences in overdose outcomes as a primary end point. Therefore, we selected a composite of evidence-based prescribing practices that are shown to be associated with reduced overdose risk as the primary outcome (with overdose as one of several secondary outcomes) [23-29].

Our secondary outcomes include the composite outcome at the 6-month follow-up; each individual component of our primary composite outcome; and several health care use measures, including all-cause and overdose-specific emergency department or inpatient visits.

### Statistical Methods

#### Randomization and Blinding

UPMC primary care practices serve as the unit of randomization, and all clinicians within a randomized practice receive the intervention assigned to that practice. We will stratify practice randomization by quartiles (25th, 50th, and 75th) of practice size based on preliminary practice data to balance the distribution of practice sizes across study arms. An additional covariate constraint on practice size will apply to further secure a balance across 3 arms in a 1:1:1 ratio due to the presence of large sites within the stratum [30,31]. The study statistician will generate randomization lists for each stratum using an SAS macro (SAS Institute) with varying block sizes. Once the randomized sequence is generated, only 1 designated member of the study team will receive the allocation list and embed it into the EHR system. Due to the nature of the intervention, treating clinicians are not blinded as they are exposed to EHR-based flags + nudges. Investigators and analysts will remain blinded to study arm assignment during outcome derivation and initial analyses using a prespecified analytic code that does not reference study arm labels. Study arm allocation will be revealed only after primary models are finalized for interpretation.

#### Sample Size and Power

We performed a priori power calculations using a simulation study designed to mirror the trial structure. The simulation incorporated 99 practices (created by regrouping the original 181 practices by size) and accounted for an unforeseen Epic system change that limited the intervention period to 4 months rather than the initially planned 6-month intervention period. The simulation used a multivariable proportional-odds linear mixed model and assumed an average of 8 eligible high-risk patients per clinical practice (range 2-16 across 1000 simulations), an intraclass correlation coefficient of 0.01, an estimated rate of the outcome from pilot analyses, and an anticipated 1– to 8–percentage point improvement in the primary composite outcome distribution from the usual care to the elevated risk flag plus nudge intervention arms, which is the a priori primary comparison for the study (Table 1). With 181 practices and a total sample size of 800 patients, the trial is estimated to have 81.3% power (α=.05) to detect the anticipated effect size between the elevated risk flag + nudges and usual care arms adjusting for a 1% attrition rate due to the development of malignant cancer or transition to hospice care after enrollment.

**Table 1:** Outcome distributions assumed for power simulations for outcomes at 4 months (n=800).

Outcome			Usual care (%)		Elevated risk flag (%)		Elevated risk flag + nudges (%)
Naloxone			12		17.3		20.7
No high dose			90		91.3		92.7
No benzodiazepine overlap			94		94.7		95.3
**Overall distribution of ordinal composite outcome**							
	Score 3	10.1		14.7		18.1	
	Score 2	76.2		74.1		72.7	
	Score 1	13.2		10.8		8.9	
	Score 0	0.7		0.6		0.5	

#### Statistical Analysis

We hypothesize that primary care providers in clinics assigned to the elevated risk flag and behavioral nudges arm will demonstrate safer prescribing behaviors than those assigned to usual care (primary hypothesis) or elevated risk flag only (secondary hypothesis) arms.

The primary analysis will follow the intention‑to‑treat principle and CONSORT 2010 statement extension [32] using the target cohort of 800 patients. Any additional patients enrolled after the Epic system change will be included in secondary and sensitivity analyses. We will analyze the primary ordinal outcome at 4 months using a mixed‑effects ordinal logistic regression model with random intercepts for practice and fixed effects for study arm (elevated risk flag and elevated risk flag + nudges vs usual care). We will adjust for baseline outcome value (for evaluating changes in outcome scores in the analysis), practice size, and any additional baseline imbalanced covariates when appropriate. Secondary pairwise hypotheses will be evaluated only if the primary contrast (ie, elevated risk flag + nudges vs usual care) is statistically significant following a gatekeeping strategy to preserve an overall α at .05.

We will analyze secondary outcomes primarily using multivariable generalized linear mixed models with appropriate link functions, variable specifications, and distributional assumptions. We will also examine “off-target” effects among all patients seen in randomized primary care clinics, including low-risk patients not subject to the alerts but treated by clinicians who had seen alerts for other patients, as part of our secondary analyses.

Sensitivity analyses will assess robustness by varying outcome definitions (ie, using only prescription fill data vs only prescription order data), altering follow-up length (6 months rather than 4 months), conducting as-treated analysis for any arm crossover, and applying alternative eligibility criteria for prior OUD. We will introduce interaction terms for randomized groups by age, sex, race and ethnicity, baseline opioid MME, history of OUD, prior overdose, and psychiatric diagnoses and assess treatment heterogeneity effects.

Missing data are expected given the use of routinely collected EHR data and variability in clinical documentation and data completeness across practices. When applying the gradient boosting model to enrolled patients, missing predictor values are addressed using simple imputation procedures, with continuous variables imputed using median values and categorical variables imputed using mode values. The ordinal primary outcome will be computed from UPMC EHR data; however, some outcome components or covariates may have missing values. We will examine missing data patterns and compare baseline characteristics between participants with and without missing outcome data. When appropriate, we will implement multiple imputation with the fully conditional specification approach [33] or use joint modeling or shared parameter model approaches that simultaneously model the outcome and dropout or death as sensitivity analyses when appropriate.

Descriptive statistics will summarize practice-, clinician-, and patient-level characteristics by group. Statistical assumptions will be evaluated for each model, with corrective procedures applied when needed. Statistical significance will be determined using a 2-sided *P* value threshold of .05. Analyses will be conducted using SAS (version 9.4) and R (version 4.0.2; R Foundation for Statistical Computing).

### Ethical Considerations

The clinical trial was approved by the University of Pittsburgh Institutional Review Board (IRB; STUDY22040068) on February 24, 2024, before trial initiation and is overseen by an independent data and safety monitoring board (DSMB). The study involves secondary analysis of limited EHR data from UPMC. In accordance with federal regulations and institutional policies, the IRB granted a waiver of patient informed consent and HIPAA (Health Insurance Portability and Accountability Act) authorization as the intervention poses minimal risk to participants and aligns with routine EHR-based clinical workflows. All analyses are conducted within the secure University of Pittsburgh Health Services Research Data Center server environment. This study complies with institutional data use agreements and all relevant guidelines and regulations for human subject research and data privacy. As this study involves secondary analysis of existing EHR data with no direct participant interaction no participant compensation was provided.

An independent institutional DSMB supported by the University of Pittsburgh Clinical and Translational Science Institute provides ongoing oversight. The DSMB held its initial meeting on December 6, 2024; reviewed the study design and charter; elected a chair; and approved the trial to begin. The DSMB is independent of the study investigators and funder and operates under a formal charter. The DSMB meets quarterly (or as needed) following trial initiation to review safety, protocol adherence, and study progress. Serious adverse events and unanticipated problems are to be reported to the DSMB chair and coordinators within 24 to 48 hours of investigator awareness and to the IRB per institutional policy. The DSMB may recommend continuation, modification, temporary suspension, or early termination of the trial.

## Results

This study was funded by the US National Institutes of Health in June 2022. Clinical trial enrollment began on March 10, 2025. Enrollment for the primary analysis cohort was completed (n=798) on May 26, 2025. Additional participants continued to be enrolled for secondary analyses (n=1662 as of December 12, 2025). Data analysis for the primary cohort began in January 2026, and results are expected by the middle of 2027.

## Discussion

We describe the protocol for a cluster randomized trial evaluating an ML-guided set of behavioral nudges embedded within the EHR to reduce overdose-related risk in primary care. We anticipate that this intervention will improve evidence-based prescribing behaviors associated with reduced overdose risks among elevated-risk patients and, in turn, reduce overdose risk. By integrating accurate risk prediction with actionable behavioral nudges, this study is expected to provide evidence on a scalable strategy to influence clinicians’ decision-making and improve opioid safety in real-world primary care settings.

Although prior studies have tested various prediction models for overdose [4,34-42] and others have examined behavioral nudging strategies to influence prescriber behavior [15,43-46], we are not aware of any prior randomized trials that test the combined impact of overdose risk prediction and behavioral nudges. Importantly, several overdose risk prediction tools have already entered clinical practice with limited validation and evaluation, underscoring the necessity of rigorous clinical trials to assess their real-world effectiveness and inform policy decisions. By testing these tools in practice in a large RCT, we will provide high-quality evidence on the effectiveness of these interventions, providing clinicians, health system leaders, and policymakers with critical data to guide adoption and implementation.

In designing the trial, we chose practice-level randomization rather than patient- or clinician-level randomization to minimize the risk of contamination across arms and ensure that all clinicians within practices received a consistent intervention. UPMC’s robust clinical informatics team and the presence of a large number of primary care practices operating on a unified EHR platform enabled this practice-level approach, allowing us to isolate the effect of the intervention alone on prescribing practices. Our months-long silent and live testing phases are a strength of the study, enabling calibration and refinement of the alerts to maximize predictive performance while minimizing excessive firing, workflow disruption, and alert fatigue. Although we anticipate that larger practices may generate a higher volume of alerts than smaller practices due to greater patient volume, our stratified randomization by practice size is designed to balance this difference across study arms. Our 3-arm design structure enables assessment of the added benefit of nudges over the risk flag alone.

There are some important potential limitations in this study. First, a challenge in designing trials addressing opioid overdose in primary care is the dynamic nature of how practices respond to the opioid epidemic and how the epidemic itself changes. For example, practices in our usual care arm could begin to adopt safer prescribing behaviors similar to those encouraged by our intervention if UPMC were to introduce parallel system-wide initiatives during the study period; however, we do not anticipate any major changes during our trial period. Other potential challenges include alert fatigue, variable clinician engagement, potential EHR system changes or updates, and model drift over time. We have mitigated these limitations through our iterative nudge design, model fairness and calibration assessments, continuous monitoring through silent and live phases, and proactive adaptation to systematic changes. Second, because overdose events are relatively rare, the trial is not powered to detect differences in overdose outcomes as a primary end point. Instead, we use a composite of prescribing behaviors with strong evidence linking them to reduced overdose risk as a pragmatic and actionable proxy [47]. Third, while we assigned study arm by practice to minimize the risk of contamination, some physicians and other clinicians may work out of multiple departments during the course of the study. Fourth, while our initial plan was for a 6-month intervention period, unexpected changes occurred in Epic within UPMC that required shortening the intervention period for the primary analysis to 4 months. However, we anticipate that most intervention effects will occur within the first four months of the trial, when clinicians receive the initial set of justification alerts. These alerts are programmed to reappear based on predefined criteria. Specifically, certain alerts will reappear 3 months after the index encounter if the underlying risk condition persists (eg, continued high opioid dosage or opioid/benzodiazepine overlap after a prior justification), whereas others reappear sooner if the triggering condition recurs (eg, a new opioid prescription). Regardless, we have preplanned a 6-month secondary outcome to compare any difference in intervention efficacy. Finally, we acknowledge that our intervention will focus on the primary care setting, and while it may generalize to other parts of the health care system that use an EHR, it will not help the many people at risk of overdose who do not engage with the health care system.

Our planned dissemination of study results includes the usual academic channels, including journal publication and presentation at scientific meetings in late 2026 and early 2027. We are also in regular communication with the clinical leadership of primary care in the health system, as well as the major informatics groups, enabling real-time dissemination of findings to interested parties who can implement changes after the clinical trial is completed. Our research team also received supplemental funding from the National Institutes of Health to compile “lessons learned” from our implementation and create a playbook that will guide others in implementing similar overdose prediction tools in the EHR in an ethical and successful manner.

In summary, the mPROVEN study is among the first pragmatic RCTs to integrate ML‑based opioid overdose risk prediction with behavioral nudges directly within a large health system EHR. By combining advances in data science, behavioral economics, and implementation science, the study aims to reduce opioid overdose risk in primary care using a scalable and low-touch intervention to address a high-priority public health issue.

## Appendix

Multimedia Appendix 1 Peer-reviewer report from CDMA - Clinical Data Management and Analysis Study Section, Healthcare Delivery and Methodologies Integrated Review Group, National Institute on Drug Abuse (National Institutes of Health, USA).

## Abbreviations

CONSORT: Consolidated Standards of Reporting Trials
DSMB: data and safety monitoring board
EHR: electronic health record
HIPAA: Health Insurance Portability and Accountability Act
IRB: Institutional Review Board
ML: machine learning
MME: morphine milligram equivalent
mPROVEN: Machine Learning Prediction and Reducing Overdoses With Electronic Health Record Nudges
OUD: opioid use disorder
RCT: randomized controlled trial

## Data Availability

The datasets generated or analyzed during this study are available from the corresponding author on reasonable request. A data use agreement, institutional and privacy approvals, and cost recovery fees for data preparation and transfer may be required.
