# Supplementary material for: Machine Learning Prediction and Reducing Overdoses With Electronic Health Record Nudges (mPROVEN) in the Primary Care Setting: Protocol for a Cluster Randomized Controlled Trial
Source: JMIR Res Protoc. 2026 May 4;15:e94007. doi: 10.2196/94007 (PMC13184600; doi:10.2196/94007)
Supplement: Multimedia Appendix 1 [file resprot_v15i1e94007_app1.pdf]

SUMMARY STATEMENT

(Privileged Communication)

PROGRAM CONTACT:
Shelley Su
301-402-3869
shelley.su@nih.gov

Release Date: 02/24/2022
Revised Date:

Application Number: 2 R01 DA044985-04
Formerly: 5R01DA044985-03

Principal Investigator

GELLAD, WALID F.

Applicant Organization: UNIVERSITY OF PITTSBURGH AT PITTSBURGH

Review Group: CDMA
Clinical Data Management and Analysis Study Section

Meeting Date: 02/10/2022
Council: MAY 2022
Requested Start: 07/01/2022

RFA/PA: PA20-183
PCC: CM/ZIS

Project Title: Machine-Learning Prediction and Reducing Overdoses with EHR Nudges (mPROVEN)
SRG Action: Impact Score:24 Percentile:2
Next Steps: Visit https://grants.nih.gov/grants/next_steps.htm
Human Subjects: 30-Human subjects involved - Certified, no SRG concerns
Animal Subjects: 10-No live vertebrate animals involved for competing appl.
Gender: 1A-Both genders, scientifically acceptable
Minority: 1A-Minorities and non-minorities, scientifically acceptable
Age: 3A-No children included, scientifically acceptable

Project Year	Direct Costs Requested	Estimated Total Cost
4		
5		
6		
7		
8		
TOTAL		

ADMINISTRATIVE BUDGET NOTE: The budget shown is the requested budget and has not been adjusted to reflect any recommendations made by reviewers. If an award is planned, the costs will be calculated by Institute grants management staff based on the recommendations outlined below in the COMMITTEE BUDGET RECOMMENDATIONS section.

2R01DA044985-04 Gellad, Walid

RESUME AND SUMMARY OF DISCUSSION: In this R01 renewal, the investigators propose to combine machine learning based overdose risk prediction tools with behavioral nudges through a scalable electronic health records (EHR) intervention to improve clinician prescribing behavior to reduce opioid overdose risk. The review committee considered the investigative team to be productive during their prior funding by their publications in scientific journals and also acquiring two additional funding to evaluate opioid prescribing risks. They considered the scientific foundation of the study to be well supported by their prior studies and by scientific literature. They thought the study to be highly significant due to existing risk prediction tools being insufficiently accurate, the proposed study if successful, could be applied in primary care practices and to additional areas of substance use disorder treatment and other health care settings. They considered the investigative team well experienced to conduct the studies, while insufficient expertise in the area of artificial intelligence. Other noted strengths include the integration of machine-learning opioid risk prediction tool into the EHR to maximize the impact of a behavioral nudge intervention to be innovative, rigorous scientific approach with strong design, use of validated machine learning models on opioid overdose, validation with three different external datasets, consideration of racial and ethnic differences, and appropriate statistical plans. However, minor concerns were noted in the rigor of the approach. These include limited discussion on handling the missing data, inclusion of only adults in the study population, and some reviewers were unclear as to why the proposed prediction tool couldn't be tested in the ongoing study. In addition, reviewers noted limited details on the composition of the data safety and monitoring plan. Following discussion, the review committee agreed the study would have a high impact in the study of opioid misuse and overdose.

DESCRIPTION: The US continues to grapple with an opioid epidemic, with ~69,700 opioid overdose deaths in 2020. Health systems have instituted multiple interventions to reduce patient risk, many focusing on decreasing unsafe opioid prescribing among those viewed as high-risk. However, there are limited tools to identify who is truly at high risk of overdose, leading to burdensome interventions targeting an overly broad population or missing key high-risk individuals. Even if those who are at risk can be identified, the interventions lack effective strategies to change clinician behavior, focusing instead on blunt tools to reduce prescribing rather than reduce risk. In prior work, we developed and externally validated machine-learning algorithms that identify patients at high risk of opioid overdose, even if not actively prescribed opioids. Separately, we demonstrated how behavioral nudge alerts embedded in the electronic health record (EHR) can be combined with risk prediction tools to change clinician behavior. In this project, we propose to reduce opioid overdose risk by bringing together machine-learning based overdose risk prediction and behavioral nudges through a scalable EHR intervention to improve clinician prescribing behavior. In a large academic health system (UPMC), we propose the following specific aims: (1) Incorporate our previously validated machine learning algorithm into the EHR to predict 3- month risk of opioid overdose; (2) Pilot test a clinician-targeted behavioral nudge intervention in the EHR for patients at high predicted risk for opioid overdose; (3) Evaluate the effectiveness of providing risk scores in the EHR with and without a behavioral nudge to improve opioid prescribing safety and reduce overdose risk. In Aim 1, we will apply our gradient boosting machine overdose prediction algorithm to the UPMC Epic-based EHR. We will optimize the algorithm for use in UPMC primary care practices, addressing model accuracy and algorithmic biases. In Aim 2, we will combine the risk score generated by our algorithm with clinician nudges in the EHR, using a 3-phase pilot with focus groups, silent testing, and live testing in 3 primary care practices. The nudge intervention will target clinicians caring for high-risk patients and will use active choice prompts for naloxone and accountable justifications for opioid and benzodiazepine prescribing. In Aim 3, we will conduct a cluster randomized trial in 45 UPMC primary care practices, with 3 arms: (1) usual care; (2) EHR-embedded risk score; 3) EHR-embedded risk score coupled with the nudge from Aim 2. The

EHR-embedded risk score arm will consist of an alert in the EHR that identifies the patient as high risk for overdose. In the risk score coupled with nudge arm, a similar EHR alert about high-risk status will flag, along with the nudges from Aim 2. The primary outcome will be a composite of 3 prescribing practices associated with reduced risk of overdose: naloxone prescription, opioid dosage <50MME per day, and no opioid/benzodiazepine overlap. Our proposal builds on our prior NIDA-funded work and experience with nudge interventions and is aligned with NIDA's strategic goals to develop and test novel strategies for preventing opioid misuse and overdose.

PUBLIC HEALTH RELEVANCE: The US continues to grapple with an opioid epidemic, with close to 70,000 opioid overdose deaths in 2020. This project will test whether combining machine-learning based overdose risk prediction tools with simple behavioral 'nudge' interventions in the electronic health record can improve clinician prescribing behavior to reduce overdose risk. If successful, the project will demonstrate how risk prediction can help target low cost and scalable interventions to reduce overdose risk, opening the door to larger scale implementation and substantial public health benefit.

CRITIQUE 1

Significance: 1
Investigator(s): 1
Innovation: 2
Approach: 3
Environment: 1

Overall Impact: This 5-year renewal R01 application follows a three year NIDA R01 grant focused on reducing death from opioid overdose through enhanced prediction and deployment of clinician "nudges" in the electronic health record. Three sequential aims that assess the performance of the gradient-boosted algorithm that incorporated EHR data for predicting opioid overdose within 3 months, pilot test a behavioral judge intervention for clinicians treating patients identified as high risk, followed by a cluster randomized, 3-arm trial to compare opioid prescribing safety among sites randomized to usual care, risk score only, or risk score plus nudge intervention. Enthusiasm for this project is very high, with only a couple of minor weaknesses in the approach identified. The investigative team has proven the ability to collaborate, and the proposed work is a natural extension of the prior funded grant, and is informed by information acquired through additional funding mechanisms. The project has very high clinical significance, given the enormity of the opioid crisis and the possibility of rapid scaling up and dissemination of the risk prediction tool and behavioral nudges within Epic EHRs. The project has the potential to establish the standard of national standard for opioid prescribing within Epic EHRs.

1. Significance:

Strengths

- Opioid overdose continues to be a major public health problem and an intervention that can enhance application of best practices to minimize risk of death from opioid overdose would be a major public health benefit.
- Existing risk prediction tools are insufficiently accurate. A more accurate/targeted risk prediction tool for opioid dose would likely have greater acceptance and uptake by clinicians.
- If successful, the risk prediction tool and behavioral nudge interventions could be rapidly disseminated and applied at scale across many health care systems.

- The results from the prior funding period provide compelling preliminary data for conducting the proposed research, with evidence of enhanced model prediction of overdose in Medicare and Medicaid datasets, which included external validation, identification of bias in the model, and demonstration of stability of risk over 3-month intervals.
- Using accountable justification and peer comparison, the investigators have previously demonstrated the substantial utility of nudges to change opioid prescribing in opiate-naïve patients.
- The investigators have previously demonstrated the ability to conduct a cluster-randomized trial in an earlier PCORI funded project.

Weaknesses

- None identified.

2. Investigator(s):

Strengths

- PI Gellad has 15 years' experience evaluating safety of prescription medications, has worked extensively with the Co-Is involved in this project, and has been highly productive in opioid prescription research.
- Co-I Arnold is a primary care physician and Director of the eRecord at UPMC and thus will be able to facilitate implementation of the algorithm.
- Co-I Kraemer is the PI of the recent cluster randomized trial on which Aim 3 of this project is based, and thus contributes the necessary trial design and management experience.
- Co-Is Lo-Ciganic and Guo have extensive machine learning and data informatics expertise.
- Additional Co-Is have expertise in vulnerable and underserved populations, pharmacoepidemiology and biostatistics.
- An expert on algorithm fairness, Dr. Ghani, will provide consultation on this aspect of the algorithm.

Weaknesses

- None identified.

3. Innovation:

Strengths

- The incorporation of naloxone and benzodiazepine co-prescribing (rather than just MME equivalents) in the behavioral nudges is a novel approach to improving opioid prescribing safety.
- The combination of enhanced risk prediction and behavioral nudges is a novel application in the area of opioid prescribing.

Weaknesses

- Minor: Several trials are underway to evaluate the utility of nudge interventions to address clinician opioid prescribing practices.

4. Approach:

Strengths

- The cluster randomized design will allow for separate evaluation of the components of the intervention for the clinical utility of the risk score versus the score plus behavioral nudges.
- Active choice to force decision making is an effective method to overcome clinician status quo bias.
- There is an extensive set of features to be used in the machine learning, with several additional features available in the EHR that should enhance the prediction model. The GBM model provides greater interpretability than deep neural networks at equivalent accuracy. Even if a simple predictive variable is superior to the algorithm, the evaluation of nudges in Aims 2 and 3 still have significant utility; consequently, aims 2 and 3 are not dependent on Aim 1.
- Model fairness will be assessed, with an emphasis on the most important metric – minimizing false negative rates.
- Cognitive burden on clinicians from the alerts will be assessed as will system usability in Aim 2.
- The cluster randomized design is the most appropriate to avoid contamination between providers and to best represent real-world implementation.
- The statistical analytic plan for Aim 3 has been carefully thought through and is appropriate.
- Co-I Arnold and a letter of support from Chief Medical Information Officer provide confidence in the team's ability to implement the algorithm into the UPMC EHR.

Weaknesses

- Minor: Overdoses that do not present to medical care or to care outside the UPMC system will be missed. However, the investigators have appropriate plans in place to address this concern.
- Minor: The algorithm will only be applied to patients with a prior history of an opioid prescription, because that was the sample used for building the original model. This is a minor concern because overdose with an initial opioid prescription is rare; risk for opioid overdose is much greater in those with repeat prescriptions.
- Minor: The statement that "the effect of missing data on results should be minimal" is not explained. It would appear that risk score calculation could be impacted by missing data, depending on the final variables included in the model, which could lead to misclassification of individuals.

5. Environment:

Strengths

- Pennsylvania is one of the hardest hit states in the opioid epidemic, making UPMC an excellent site to conduct the proposed RCT.
- The UPMC database is large enough to have high confidence that the project will be adequately powered.

Weaknesses

- None identified.

Study Timeline

Strengths

- Aim 1 will occur in Year 1; Aim 2 in Year 2. This leaves Years 3 and 4 to conduct Aim 3, the randomized trial, with the final year of the grant for analysis. Given that "recruitment" is simply a passive exercise of identifying high risk patients through routine primary care visits, and the power analysis indicate excellent probability of identifying the required number of high risk subjects, this timeline is appropriate.

Weaknesses

- None identified.

Protections for Human Subjects

Acceptable Risks and/or Adequate Protections

- Protections in Aims 1 and 2 are straightforward. The greatest potential risk in Aim 1 is not to individuals but for the implications for patients with sensitive attributes. However, a plan is in place to evaluate the algorithm for bias and adjust for fairness. For Aim 3, the investigators identify that the trial does not present more than minimal risk, which is justified. Investigators will be free to apply or not apply the behavioral nudges as they see fit, so patient care should not be compromised by the design of the trial, and is likely to be enhanced.

Data and Safety Monitoring Plan (Applicable for Clinical Trials Only):

Unacceptable

- The composition of the DSMB is not specified (specialties of the board members, and nature of data (blinded, unblinded) to be presented to the board.

Inclusion Plans

- Sex/Gender: Distribution justified scientifically
- Race/Ethnicity: Distribution justified scientifically
- For NIH-Defined Phase III trials, Plans for valid design and analysis: Scientifically acceptable
- Inclusion/Exclusion Based on Age: Distribution justified scientifically
- Only adults are included because that population is whom the prior work was based on.

Vertebrate Animals

Not Applicable (No Vertebrate Animals)

Biohazards

Not Applicable (No Biohazards)

Renewal

- The original funding period was for three years. The investigators published six papers relevant to the project aims, with three more currently under review at the time of submission. They also filed for one patent related to machine learning and predicting opioid use disorder and overdose and acquired two additional grants to further evaluate opioid prescribing risks.

Resource Sharing Plans

Acceptable

Budget and Period of Support

Recommend as Requested

CRITIQUE 2

Significance: 1
Investigator(s): 2
Innovation: 2
Approach: 1
Environment: 1

Overall Impact: The aim of this study is to use machine learning methods and data for Medicaid beneficiaries in two states to develop algorithms to predict who is at risk of problematic prescription opioid use and overdose. The overall impact of the proposed study is high. The study team has the expertise and the environment to achieve the study aims. The resubmission has addressed most of the previous critiques. Additionally, the proposed project will be using previously developed models and datasets that are readily available and have already been used in prior studies.

1. Significance:

Strengths

- Scientific premise is strong.
- Rigor of study is established by appropriate literature review
- Leveraging on existing academic-state partnerships, the project will have access to administrative claims data for all Medicaid beneficiaries in Pennsylvania and Arizona.

Weaknesses

- None noted by reviewer.

2. Investigator(s):

Strengths

- Research team consist of investigators that have expertise in health equity, pharmaco-epidemiology and safety of prescription drug use and opioid therapy.
- The study team has a history of collaboration in this field.

Weaknesses

- None noted by reviewer.

3. Innovation:

Strengths

- While the methods are not innovative, the application to this clinical domain is novel.
- The use of claims plus clinical data is innovative.

Weaknesses

- The methods used are not innovative.

4. Approach:

Strengths

- The scientific approach is rigorous with a strong design. Conceptual framework for the entire project is well described.
- The proposed methods account for potential sex, racial and ethnic differences in prediction.

Weaknesses

- None identified

5. Environment:

Strengths

- The university has robust machine learning infrastructure.
- The team has the existing physical resources necessary to successfully complete the proposed study.

Weaknesses

- None noted by reviewer.

Study Timeline

Strengths

- The timeline is appropriate to complete the study.

Weaknesses

- None noted by reviewer.

Protections for Human Subjects

Acceptable Risks and/or Adequate Protections

- Researchers have addressed the relevant human subjects' concerns.

Data and Safety Monitoring Plan (Applicable for Clinical Trials Only):

Appropriate

Inclusion Plans

- Sex/Gender: Distribution justified scientifically
- Race/Ethnicity: Distribution justified scientifically

- For NIH-Defined Phase III trials, Plans for valid design and analysis: Not applicable
- Inclusion/Exclusion Based on Age: Distribution justified scientifically
- The distribution is appropriate for the study population.

Vertebrate Animals
No, animal welfare concerns or incomplete

Biohazards
Not Applicable (No Biohazards)

Budget and Period of Support
Recommend as Requested

CRITIQUE 3

Significance: 1
Investigator(s): 4
Innovation: 4
Approach: 1
Environment: 1

Overall Impact: The renewal application is significant in testing the ML models that have been developed and tested by the research team via randomized trials. The application holds medium to high impact to demonstrate the impact of ML in clinical decision support and adoption of ML in clinical practice. The application show strengths in its significance, methods, and environment. There are minor concerns that may have an impact on the successful completion of the proposal. Innovation is not adequately discussed and many of the mentioned could be part of the ongoing R01 instead of the renewal. ML expertise is not strong or sufficient in the PI and Co-Is to support the AIMs.

1. Significance:
Strengths
- Opioid overdose is a priority of research.
- Offering ML prediction tools to identify who is truly at risk of overdose.
- Incorporated the investigation team's ML models into EHR to rigorously test the applications via randomized trials.

Weaknesses
- None noted by reviewer.

2. Investigator(s):
Strengths

- The PI has a long term collaboration with the Co-Is.
- The team has complementary and integrated expertise to conduct the renewal application.

Weaknesses

- It is not an easy task to identify any team leaders have ML training and expertise to lead AIM1
- Dr. Ghani, a consultant of ML, may not be sufficient for the proposed AIMs.

3. Innovation:

Strengths

- Applying ML that has been developed in the ongoing R01 to claims and clinical data is a strength. The innovation was recognized in the ongoing R01
- Evaluating the effectiveness of risk scores in EHR via a cluster randomized trial in 45 UPMC primary care practices.
- Rigorously testing of predictive models in randomized trials is a must to demonstrate the clinical effectiveness.

Weaknesses

- The ML method itself is not innovative and the innovation in the renewal application is unclear.
- The integration of ML prediction tool could be tested in the ongoing R01 as an integrated project instead of a separate R01 with less novelty.

4. Approach:

Strengths

- Current R01 has developed and validated ML prediction models on opioid overdose with external validation over 3 datasets.
- The team has conducted a randomized trial of 78 oncologists and 14,607 cancer patients.
- The use of nudges is based upon a conceptual framework

Weaknesses

- None noted by reviewer.

5. Environment:

Strengths

- All participating institutions are supportive in scientific environment.

Weaknesses

- None noted by reviewer.

Study Timeline

Strengths

- Acceptable

Protections for Human Subjects
Acceptable Risks and/or Adequate Protections

Inclusion Plans

- Sex/Gender: Distribution justified scientifically
- Race/Ethnicity: Distribution justified scientifically
- For NIH-Defined Phase III trials, Plans for valid design and analysis: Scientifically acceptable
- Inclusion/Exclusion Based on Age: Distribution justified scientifically

Vertebrate Animals
Not Applicable (No Vertebrate Animals)

Biohazards
Not Applicable (No Biohazards)

Renewal

- very productive team

Resource Sharing Plans
Acceptable

Budget and Period of Support
Recommend as Requested

THE FOLLOWING SECTIONS WERE PREPARED BY THE SCIENTIFIC REVIEW OFFICER TO SUMMARIZE THE OUTCOME OF DISCUSSIONS OF THE REVIEW COMMITTEE, OR REVIEWERS' WRITTEN CRITIQUES, ON THE FOLLOWING ISSUES:

PROTECTION OF HUMAN SUBJECTS: ACCEPTABLE

INCLUSION OF WOMEN PLAN: ACCEPTABLE

INCLUSION OF MINORITIES PLAN: ACCEPTABLE

INCLUSION ACROSS THE LIFESPAN: ACCEPTABLE

COMMITTEE BUDGET RECOMMENDATIONS: The budget was recommended as requested.

Footnotes for 2 R01 DA044985-04; PI Name: Gellad, Walid F.

NIH has modified its policy regarding the receipt of resubmissions (amended applications).See Guide Notice NOT-OD-18-197 at https://grants.nih.gov/grants/guide/notice-files/NOT-OD-18-197.html. The impact/priority score is calculated after discussion of an application by averaging the overall scores (1-9) given by all voting reviewers on the committee and multiplying by 10. The criterion scores are submitted prior to the meeting by the individual reviewers assigned to an application, and are not discussed specifically at the review meeting or calculated into the overall impact score. Some applications also receive a percentile ranking. For details on the review process, see http://grants.nih.gov/grants/peer_review_process.htm#scoring.

MEETING ROSTER

Clinical Data Management and Analysis Study Section
Healthcare Delivery and Methodologies Integrated Review Group
CENTER FOR SCIENTIFIC REVIEW
CDMA
02/10/2022 - 02/11/2022

Notice of NIH Policy to All Applicants: Meeting rosters are provided for information purposes only. Applicant investigators and institutional officials must not communicate directly with study section members about an application before or after the review. Failure to observe this policy will create a serious breach of integrity in the peer review process, and may lead to actions outlined in NOT-OD-14-073 at https://grants.nih.gov/grants/guide/notice-files/NOT-OD-14-073.html, NOT-OD-15-106 at https://grants.nih.gov/grants/guide/notice-files/NOT-OD-15-106.html, and NOT-OD-18-115 at https://grants.nih.gov/grants/guide/notice-files/NOT-OD-18-115.html, including removal of the application from immediate review.

CHAIRPERSON(S)

PAYNE, PHILIP R O, PHD
PROFESSOR AND DIRECTOR
INSTITUTE FOR INFORMATICS
SCHOOL OF MEDICINE
WASHINGTON UNIVERSITY
ST LOUIS, MO 63110

MEMBERS

AGU, EMMANUEL, PHD *
PROFESSOR
DEPARTMENT OF COMPUTER SCIENCE
WORCESTER POLYTECHNIC INSTITUTE
WORCESTER, MA 01609

ARNAOUT, RIMA, MD
ASSOCIATE PROFESSOR
DEPARTMENT OF MEDICINE
SCHOOL OF MEDICINE
UNIVERSITY OF CALIFORNIA, SAN FRANCISCO
SAN FRANCISCO, CA 94117

BECKETT, LAUREL A, PHD
DISTINGUISHED PROFESSOR EMERITA
DEPARTMENT OF PUBLIC HEALTH SCIENCES
SCHOOL OF MEDICINE
UNIVERSITY OF CALIFORNIA, DAVIS
DAVIS, CA 95616

BIAN, JIANG, PHD
ASSOCIATE PROFESSOR
DEPARTMENT OF HEALTH OUTCOMES
AND BIOMEDICAL INFORMATICS
COLLEGE OF MEDICINE
UNIVERSITY OF FLORIDA
GAINESVILLE, FL 32610

CANAHUATE, GUADALUPE, PHD
ASSOCIATE PROFESSOR
DEPARTMENT OF ELECTRICAL AND COMPUTER
ENGINEERING
UNIVERSITY OF IOWA
IOWA CITY, IA 52242

CANNESSON, MAXIME, PHD
PROFESSOR AND CHAIR
DEPARTMENT OF ANESTHESIOLOGY
AND PERIOPERATIVE MEDICINE
UNIVERSITY OF CALIFORNIA, LOS ANGELES
LOS ANGELES, CA 90095

CATO, KENRICK DWAIN, PHD
ASSISTANT PROFESSOR
DEPARTMENT OF EMERGENCY MEDICINE
SCHOOL OF NURSING
COLUMBIA UNIVERSITY
NEW YORK, NY 10033

CHHABRA, NEERAJ, MD *
ASSISTANT PROFESSOR
DEPARTMENT OF EMERGENCY MEDICINE
COOK COUNTY HEALTH
CHICAGO, IL 60612

CHIDAMBARAN, VIDYA, MD *
PROFESSOR
DEPARTMENT OF ANESTHESIA
CINCINNATI CHILDREN'S HOSPITAL
CINCINNATI, OH 45229

DUNLOP, BOADIE W, MD
PROFESSOR
DEPARTMENT OF PSYCHIATRY AND BEHAVIORAL
SCIENCES
SCHOOL OF MEDICINE
EMORY UNIVERSITY
ATLANTA, GA 30329

ELHABIAN, SHIREEN YOUSSEF, PHD *
RESEARCH ASSISTANT PROFESSOR
SCHOOL OF COMPUTING
THE UNIVERSITY OF UTAH
SALT LAKE CITY, UT 84112

FRANCESCHINI, NORA, MD, MPH *
PROFESSOR
DEPARTMENT OF EPIDEMIOLOGY
UNIVERSITY OF NORTH CAROLINA AT CHAPEL HILL
CHAPEL HILL, NC 27514

GOLDSTEIN, BENJAMIN ALAN, MPH, PHD *
ASSOCIATE PROFESSOR
DEPARTMENT OF BIOSTATISTICS AND BIOINFORMATICS
DUKE UNIVERSITY
DURHAM, NC 27705

GONG, YANG, PHD, MD *
ASSOCIATE PROFESSOR
SCHOOL OF BIOMEDICAL INFORMATICS
UNIVERSITY OF TEXAS
HEALTH SCIENCE CENTER AT HOUSTON
HOUSTON, TX 77030

GRANDO, MARIA ADELA, PHD *
ASSOCIATE PROFESSOR
BIOMEDICAL INFORMATICS, COLLEGE OF HEALTH
SOLUTION
COLLEGE OF MEDICINE MAYO CLINIC
ARIZONA STATE UNIVERSITY
PHOENIX, AZ 85008

HERNANDEZ-BOUSSARD, TINA, PHD
ASSOCIATE PROFESSOR OF MEDICINE
BIOMEDICAL DATA SCIENCE AND SURGERY
SCHOOL OF MEDICINE
STANFORD UNIVERSITY
STANFORD, CA 94305

HIMES, BLANCA E, PHD
ASSOCIATE PROFESSOR
DEPARTMENT OF BIOSTATISTICS, EPIDEMIOLOGY
AND INFORMATICS
PERELMAN SCHOOL OF MEDICINE
UNIVERSITY OF PENNSYLVANIA
PHILADELPHIA, PA 19104

HORVITZ-LENNON, MARCELA V, MD, MPH
PROFESSOR, SENIOR PHYSICIAN SCIENTIST
RAND CORPORATION
BOSTON, MA 02116

KENNEDY, RICHARD E, MD, PHD
ASSOCIATE PROFESSOR
DEPARTMENT OF MEDICINE
UNIVERSITY OF ALABAMA
BIRMINGHAM, AL 35294

KHAN, BILAL, PHD
PROFESSOR OF DATA SCIENCE
DEPARTMENTS OF COMMUNITY &
GLOBAL HEALTH AND COMPUTER SCIENCE
LEHIGH UNIVERSITY
BETHLEHEM, PA 18015

KHARRAZI, HADI, PHD, MD, MHI *
ASSOCIATE PROFESSOR
DEPARTMENT OF HEALTH POLICY AND MANAGEMENT
DIVISION OF GENERAL INTERNAL MEDICINE
SCHOOL OF MEDICINE
JOHNS HOPKINS UNIVERSITY
BALTIMORE, MD 21205

KOLECK, THERESA ANN, PHD *
ASSISTANT PROFESSOR
HEALTH PROMOTION AND DEVELOPMENT
SCHOOL OF NURSING
UNIVERSITY OF PITTSBURGH
PITTSBURGH, PA 15261

LAITEERAPONG, NEDA, MD
ASSOCIATE PROFESSOR
DEPARTMENTS OF MEDICINE AND PSYCHIATRY
AND BEHAVIORAL NEUROSCIENCE
UNIVERSITY OF CHICAGO
CHICAGO, IL 60637

LI, LANG, PHD
PROFESSOR AND CHAIR
DEPARTMENT OF BIOMEDICAL INFORMATICS
COLLEGE OF MEDICINE
THE OHIO STATE UNIVERSITY
COLUMBUS, OH 43210

LIU, MEI, PHD *
ASSOCIATE PROFESSOR
DIVISION OF MEDICAL INFORMATICS
DEPARTMENT OF INTERNAL MEDICINE
UNIVERSITY OF KANSAS MEDICAL CENTER
KANSAS CITY, KS 66160

LIU, VINCENT, MD *
RESEARCH SCIENTIST AND REGIONAL DIRECTOR
HOSPITAL ADVANCED ANALYTICS
DIVISION OF RESEARCH
KAISER FOUNDATION RESEARCH INSTITUTE
OAKLAND, CA 94612

MATHENY, MICHAEL E, MD *
ASSOCIATE PROFESSOR
ASSOCIATE PROFESSOR OF BIOMEDICAL INFORMATICS
ASSOCIATE PROFESSOR OF BIOSTATISTICS
GRECC, NASHVILLE VA MEDICAL CENTER
VANDERBILT UNIVERSITY MEDICAL CENTER
NASHVILLE, TN 37240

MCMILLAN, ALAN BLAIR, PHD *
ASSOCIATE PROFESSOR
DEPARTMENT OF RADIOLOGY
UNIVERSITY OF WISCONSIN–MADISON
MADISON, WI 53706

POPESCU, MIHAIL, PHD *
PROFESSOR
DEPARTMENT OF HEALTH MANAGEMENT AND
INFORMATICS
UNIVERSITY OF MISSOURI SCHOOL OF MEDICINE
COLUMBIA, MO 65212

RADIVOJAC, PREDRAG, PHD *
PROFESSOR
KHOURY COLLEGE OF COMPUTER SCIENCES
NORTHEASTERN UNIVERSITY
BOSTON, MA 02115

RANGANATH, RAJESH, PHD *
ASSISTANT PROFESSOR
COMPUTER SCIENCE
COURANT INSTITUTE OF MATHEMATICAL SCIENCE
NEW YORK UNIVERSITY
NEW YORK, NY 10011

RODIN, ANDREI, PHD *
ASSOCIATE PROFESSOR
DEPARTMENT OF COMPUTATIONAL AND
QUANTITATIVE MEDICINE
CITY OF HOPE
DUARTE, CA 91010

RODRIGUEZ, MANUEL, PHD *
PROFESSOR
DEPARTMENT OF COMPUTER SCIENCE AND ENGINEERING
UNIVERSITY OF PUERTO RICO
MAYAGUEZ, PR 00681-9000

SOHN, SUNGHWAN, PHD *
ASSOCIATE PROFESSOR
DEPARTMENT OF ARTIFICIAL INTELLIGENCE
AND INFORMATICS
MAYO CLINIC
ROCHESTER, MN 55905

VULLIKANTI, ANIL, PHD *
PROFESSOR
DEPARTMENT OF COMPUTER SCIENCE
AND BIOCOMPLEXITY INSTITUTE
UNIVERSITY OF VIRGINIA
CHARLOTTESVILLE, VA 22904

WANG, DONGMEI, PHD
PROFESSOR
DEPARTMENT OF BIOMEDICAL ENGINEERING
GEORGIA CANCER COALITION
GEORGIA INSTITUTE OF TECHNOLOGY
ATLANTA, GA 30332

YANG, YANG, PHD *
ASSISTANT PROFESSOR
BIOMEDICAL ENGINEERING AND IMAGING INSTITUTE
ICAHN SCHOOL OF MEDICINE AT MOUNT SINAI
NEW YORK, NY 11369

ZHANG, RUI, PHD *
ASSOCIATE PROFESSOR
DEPARTMENT OF PHARMACEUTICAL CARE & HEALTH
SYSTEMS, AND INSTITUTE FOR HEALTH INFORMATICS
UNIVERSITY OF MINNESOTA
MINNEAPOLIS, MN 55455

ZHAO, LILI, PHD *
RESEARCH ASSOCIATE PROFESSOR
DEPARTMENT OF BIOSTATISTICS
UNIVERSITY OF MICHIGAN
ANN ARBOR, MI 48109

SCIENTIFIC REVIEW OFFICER

SHIVAKUMAR, CHITTARI V, PHD
SCIENTIFIC REVIEW OFFICER
NATIONAL INSTITUTES OF HEALTH
CENTER FOR SCIENTIFIC REVIEW
BETHESDA, MD 20892

EXTRAMURAL SUPPORT ASSISTANT

JONES, BELINDA
EXTRAMURAL SUPPORT ASSISTANT
CENTER FOR SCIENTIFIC REVIEW
NATIONAL INSTITUTES OF HEALTH
BETHESDA, MD 20892

* Temporary Member. For grant applications, temporary members may participate in the entire meeting or may review only selected applications as needed.

Consultants are required to absent themselves from the room during the review of any application if their presence would constitute or appear to constitute a conflict of interest.
